# Impact of diuretics on the urate lowering therapy in patients with gout: analysis of an inception cohort

**DOI:** 10.1186/s13075-018-1559-2

**Published:** 2018-03-22

**Authors:** Laura Ranieri, Carolina Contero, Maria-Luisa Peral, Irene Calabuig, Pedro Zapater, Mariano Andres

**Affiliations:** 1 Rheumatology Deparment, Hospital General Universitario de Alicante-ISABIAL, Pintor Baeza 12, 03010 Alicante, Spain; 2 Universidad Miguel Hernández, Elche, Alicante, Spain; 3 Clinical Pharmacology Deparment, Hospital General Universitario de Alicante-ISABIAL, Alicante, Spain

**Keywords:** Gout, Diuretics, Furosemide, Thiazide, Serum urate, Allopurinol, Febuxostat

## Abstract

**Background:**

Diuretics have been associated with impaired response and refractoriness in gout, but whether this effect is still present with new urate-lowering drugs (ULD) and treat-to-target strategies is unknown. The aim of the present study was to assess the impact of the diuretics on the response to ULD in patients with gout.

**Methods:**

This was a retrospective analysis of an inception cohort. Participants were classified according to the type of ULD prescribed. We analysed the maximal dose of ULD (primary outcome variable), serum urate (SU) reduction, and the achievement of different SU targets (6 mg/dL, 5 mg/dL, and 4 mg/dL), according to the type of ULD prescribed and use of diuretics (loop and/or thiazide). We adjusted for confounders using multiple linear regression analysis.

**Results:**

We included 245 patients: 208 treated with allopurinol (66 on diuretics, 31.7%), 35 with febuxostat (19 on diuretics, 57.6%), and 2 with benzbromarone. Significantly fewer participants in the allopurinol plus diuretics subgroup achieved SU levels of less than 5 mg/dL, but we found no other significant differences in SU targets associated with diuretics. Regarding the maximum ULD dose, a simple linear regression suggested an inverse relationship with diuretics (beta = − 0.125, *p* = 0.073), but this did not hold in the multivariable analysis (beta = − 0.47, *p* = 0.833). There was no association with febuxostat (beta = − 0.116, *p* = 0.514).

**Conclusion:**

Diuretics do not appear to have a significant impact on managing gout.

**Electronic supplementary material:**

The online version of this article (10.1186/s13075-018-1559-2) contains supplementary material, which is available to authorized users.

## Background

Gout is the result of monosodium urate (MSU) crystal deposition in joints and periarticular tissues. It has become a major health issue with an increasing prevalence, affecting 0.7% to 3.7% of the population worldwide and as many as 5.9% of men in the USA [1]. It is the most common form of arthritis in western countries and is associated with significant morbidity [2], mortality [3] and health system costs [4]. Fortunately, proper management can stop flare-ups and achieve crystal dissolution and cure [5].

Hyperuricaemia, or the increase in serum urate (SU) levels, is a necessary condition for the development of gout. In the kidney, SU is easily filtered through the glomeruli and undergoes active reabsorption and secretion in the proximal convoluted tubule, with around 8% to 10% of the initially filtered SU being excreted [6]. Thus, disorders that impair the glomerular filtration of uric acid (such as renal failure) or interfere with its tubular transport (such as insulin-resistant states or drugs) would contribute to the development of hyperuricaemia and gout. As a result of this link, renal failure and diuretics are commonly seen in patients with gout [7].

Chronic kidney disease (CKD) has an impact on gout management by limiting the dosage or hampering the use of urate-lowering drugs (ULD), colchicine, and non-steroidal anti-inflammatory agents [8]. The frequent prescription of diuretics might also impact outcomes in these patients [9]. Diuretics (both loop agents and thiazides) can increase SU levels but also interfere with allopurinol, enhancing the renal clearance of its active metabolite oxypurinol and increasing the dose needed to ensure effectiveness [10]. This would also apply to advanced heart failure or hypertension, where diuretics are often used. Febuxostat is used even in patients with advanced CKD based on its hepatic metabolism, though whether diuretics modify its urate-lowering effect is unknown. If not, its use would be preferential in patients with CKD or heart failure who are on diuretics, especially when diuretics cannot be discontinued.

In this context, there is a need to determine the impact of diuretics on gout management in clinical practice, especially after the incorporation of new ULD and treat-to-target strategies [11]. This knowledge could inform management plans for patients with gout who are taking diuretics, helping to achieve suitable SU reductions that ensure the cure of the disease. The aim of the present study was to assess the impact of diuretic therapy on the response to ULD in patients with gout, according to the type of drug prescribed.

## Methods

We performed a retrospective analysis on an inception cohort in the Rheumatology Unit of the Hospital General Universitario de Alicante (Spain). The characteristics of the cohort have been described elsewhere [12]. Briefly, recruited patients are those presenting to the unit for the first time with crystal-proven gout without having received previous specialised care. The ethics committee of the Hospital General Universitario de Alicante (2017/05 Act) and the Spanish drugs regulatory administration approved this study; according to these organisations, informed consent was not necessary due to the retrospective nature of the study.

The study period for the present work was January 2014 to July 2017. All enrolled patients in the cohort were eligible for the analysis, which was based on a review of patients’ electronic clinical and pharmacy records. We excluded patients without SU results during follow up.

### Study variables

Our primary outcome variable was the maximum dose of ULD prescribed, as we considered this the best indicator of the potential influence of covariables - such as diuretics - in achieving proper SU reduction and the recommended SU targets [5] for managing patients with gout. Other outcome variables were (a) SU, both at baseline and under ULD at its maximum dose; (b) SU reduction achieved; and (c) the achievement of different SU targets (6 mg/dL; 5 mg/dL; 4 mg/dL).

The primary explanatory variable was the use and dosage of diuretic therapy, specifically loop agents (furosemide, torasemide) and thiazides, both at baseline and after achieving maximal dose of ULD. We also recorded the reason for prescribing diuretics (hypertension, heart failure, CKD, others, or mixed). Other explanatory variables were demographic (age, sex); clinical (hypertension, diabetes mellitus, dyslipidaemia, smoking, cardiovascular disease); anthropometric data (weight, height, body mass index (BMI)); laboratory measurements (estimated glomerular filtration rate (eGFR), according to the Chronic Kidney Disease Epidemiology Collaboration (CKD-EPI) formula [13]) at baseline and after maximal dose of ULD; and gout-related data (years since first attack, numbers of attacks suffered, number of involved joints, joint pattern at presentation (monoarticular, oligoarticular, polyarticular), tophi, and type of ULD employed and prior usage).

### Statistical analysis

We performed a descriptive analysis of the sample, expressing continuous variables as means (standard deviation (SD)) or medians (interquartile range ([IQR)), depending on the data distribution, and we expressed qualitative variables as frequencies and percentages. Based on clinical experience, we deemed a unified analysis of the use of diuretics inappropriate, as the patients eligible in clinics for certain ULDs (allopurinol, febuxostat, benzbromarone) are usually very heterogeneous, as are their pharmacodynamics and kinetics. To justify this division of the study population into subgroups based on the type of ULD prescribed, we compared explanatory variables using Student’s *t* test, Fisher’s exact test, and the chi-squared test.

After that, we used Student’s *t* test, the Mann-Whitney U test, and the chi-squared test to assess the association between the primary explanatory variable (use of diuretics) and the different outcome variables, stratifying analyses by type of ULD. We considered that a patient was on diuretics when being treated at the moment of maximal dosage of ULD for at least a week, to avoid bias due to diuretic start or discontinuation during follow up. Manifest variability in the type and dosage of diuretics precluded subgroup analyses. In the case of significant results in the univariable analysis, we built a linear regression model for the primary outcome (maximal dose of ULD prescribed) to adjust for potential confounders (age, sex, hypertension, diabetes mellitus, dyslipidaemia, tobacco use, cardiovascular disease, BMI, baseline SU levels and eGFR, and final eGFR). All statistical analyses were performed using SPSS 22.0 software (IBM Statistics, Armonk, NY). Statistical significance was established at *p* < 0.05.

## Results

Of 296 patients enrolled in the inception cohort, follow-up data were available for 245 patients (82.8%) with a median follow up of 8 months (IQR 4–13). General baseline characteristics are shown in Table 1; these are typical of patients with gout seen in specialised clinical practice. Median disease duration at enrolment was 4 years (IQR 0–10), with a median of 4 reported flares (IQR 2–10) having involved a median of 3 joints (IQR 2.0–4.8). At presentation, 75.1% of patients suffered from a monoarticular flare, 19.2% from an oligoarticular flare, and 5.3% from a polyarticular flare. Tophi were recorded in 23.3% of patients.

**Table 1:** Baseline characteristics of enrolled patients and group comparison according to the type of ULD used

	Whole sample (*n* = 245)	Allopurinol (*n* = 208)	Febuxostat (*n* = 35)	*P*
Age (years), mean (SD)	63.5 (13.5)	62.1 (13.2)	72.2 (11.8)	**< 0.001**
Male	212 (86.5)	189 (90.9)	22 (62.9)	**< 0.001**
HT	169 (69.0)	137 (65.9)	30 (85.7)	**0.019**
DM	60 (24.5)	47 (22.6)	13 (37.1)	0.065
DL	128 (52.2)	107 (51.4)	20 (57.1)	0.532
Smoking background (*n* = 234)	140 (59.9)	128 (64.3)	12 (36.4)	**0.006**
CVD	61 (24.9)	45 (21.6)	16 (45.7)	**0.002**
Diuretic therapy	109 (44.5)	86 (41.3)	23 (65.7)	**0.007**
Type of diuretics (*n* = 243)				**0.005**
Thiazides	61 (25.1)	51 (24.5)	10 (28.6)	
Loop agents	46 (18.9)	33 (15.9)	13 (37.1)	
Combination of diuretics	11 (4.5)	7 (3.4)	4 (11.4)	0.057
BMI (kg/m^2^), mean (SD)	29.9 (5.2)	30.0 (5.0)	30.2 (6.0)	0.790
eGFR (mL/min), mean (SD) (n = 243)	71.5 (26.4)	76.6 (23.4)	40.8 (23.0)	**< 0.001**
Chronic kidney disease (*n* = 241)	76 (31.5)	47 (22.8)	29 (82.9)	**< 0.001**
SU (mg/dL), mean (SD)	8.3 (1.7)	8.2 (1.4)	9.3 (2.4)	**0.008**
Prior use of ULD (n = 243)	58 (23.9)	50 (24.1)	8 (22.9)	0.914

Data shown as number (percentage) unless otherwise specified. In bold, statistically significant differences between allopurinol and febuxostat groups. *BMI* body mass index, calculated as weight: (height)^2^, *CVD* cardiovascular disease, *DL* dyslipidaemia, *DM* diabetes mellitus, *HT* hypertension, *SD* standard deviation, *SU* serum urate, *ULD* urate-lowering drugs

At baseline, 109 patients (44.5%) were on diuretics: 63.0% of these for an indication of hypertension, 12.6% for heart failure, 5.0% for CKD, and 19.3% for combined/other causes. More patients were taking thiazides than loop agents (57.0% versus 43.0%), and 17 (5.7%) patients were taking both. When the maximal dose of ULD was reached, 90 patients were on diuretics (36.7%); these had been stopped in 25 cases (22.9%) and started in 6 (4.4%). At this time point, more patients were taking loop diuretics than thiazides (55.5% versus 44.6%); 8 (3.3%) were on combined diuretic therapy. The supplementary material describes dosage at both time points (Additional file 1: Table S1).

With regard to ULDs, 208 patients (84.9%) were on allopurinol; 35 (14.3%) were on febuxostat; and 2 (0.8%) were on benzbromarone (excluded from the analysis). Table 1 compares baseline characteristics according to ULD treatment: the febuxostat group was significantly older, included more women, and had a higher prevalence of hypertension, CKD, and cardiovascular disease. On the other hand, there were fewer smokers, and their blood tests revealed higher levels of SU and lower eGFR. Given the significant heterogeneity, we did not pool groups but report results separately according to type of ULD.

### Patient outcomes: allopurinol group

Table 2 shows the outcomes in the 208 patients receiving allopurinol (*n* = 66, or 32.7% also on diuretics). The most frequently prescribed diuretic was hydrochlorothiazide (*n* = 37, 56.0%) at either 12.5 mg/day (*n* = 19) or 25 mg/day (*n* = 13); 8 patients (12.1%) were on combined diuretic therapy. Despite higher final SU levels in those taking diuretics compared to those who were not, the average SU reduction was similar between subgroups. We did not observe significant differences in the achievement of the different SU targets, except for SU of less than 5 mg/dL, with lower rates in the diuretic subgroup.

**Table 2:** Outcomes of the allopurinol group and comparisons on the use of diuretics

	No diuretics (*n* = 142 (68.3%))	Diuretics (n = 66 (31.7%))	P
Primary outcome variable			
Maximum dose of allopurinol (mg/day)	304.9 (120.6)	275.8 (119.7)	0.109
Secondary outcome variables			
Baseline SU (mg/dL)	8.1 (1.4)	8.4 (1.6)	0.116
Final SU (mg/dL)	4.9 (1.3)	5.4 (1.7)	**0.015**
Final eGFR (mL/min)	83.0 (24.4)	64.6 (21.5)	**< 0.001**
SU reduction (mg/dL)	3.2 (1.8)	3.0 (1.9)	0.474
Achievement of SU targets			
- SU <6 mg/dL, n (%)	110 (79.1)	47 (68.1)	0.082
- SU <5 mg/dL, n (%)	75 (54.0)	27 (39.1)	**0.044**
- SU <4 mg/dL, n (%)	34 (24.5)	11 (15.9)	0.160

Data shown as mean (SD) unless specified otherwise. In bold, statistically significant differences between subgroups. *eGFR* estimated glomerular filtration rate, *SD* standard deviation, *SU* serum urate

The maximum doses of allopurinol were comparable between subgroups, though they tended to be lower in those on diuretics and especially in those on diuretic combination; there was no difference between thiazides and loop agents (Additional file 1: Table S2). The simple linear regression analysis also suggested inverse correlation between maximum dose of allopurinol and use of diuretics (beta = − 0.125, 95% CI − 67.7 to 3.0, *p* = 0.073). However, this association was lost after adjusting for covariates (beta = − 4.70, 95% CI − 48.3 to 38.9, *p* = 0.833).

### Patient outcomes: febuxostat group

Table 3 shows the results of patients on febuxostat. Of the 33 patients, 19 (57.6%) were on diuretics. Here, the use of furosemide predominated (*n* = 13 (68.4%)) at doses of 40 mg/day (*n* = 5), 60 mg/day (n = 1), 80 mg/day (*n* = 2), and 120 mg/day (*n* = 3). Two patients (10.5%) were on combination therapy. We did not observe significant differences in the reduction of SU levels or the achievement of the different SU targets.

**Table 3:** Outcomes of the febuxostat group and comparisons on the use of diuretics

	No diuretics (n = 14 (42.4%))	Diuretics (n = 19 (57.6%))	P
Primary outcome variable			
Maximum dose of febuxostat (mg/day)	80.0 (70.0–80.0)	80.0 (40.0–80.0)	0.398
Secondary outcome variables			
Baseline SU (mg/dL)	8.7 (7.6–9.6)	9.6 (8.0–11.4)	0.174
Final SU (mg/dL)	3.5 (2.5–4.9)	3.9 (3.4–5.1)	0.439
Final eGFR (ml/min)	49.1 (37.3–69.1)	30.5 (19.0–42–9)	**0.004**
SU reduction (mg/dL)	5.0 (2.5–5.6)	4.5 (3.9–6.3)	0.733
Achievement of SU targets			
- SU <6 mg/dl, n (%)	11 (78.6)	17 (81.0)	1.000
- SU <5 mg/dl, n (%)	11 (78.6)	14 (66.7)	0.445
- SU <4 mg/dl, n (%)	8 (57.1)	11 (52.4)	0.782

Data shown as median (IQR) unless specified otherwise. In bold, statistically significant differences between subgroups. *eGFR* estimated glomerular filtration rate, *SU* serum urate

The average maximum dose of febuxostat was 80 mg/day in both subgroups, with no association with the type of diuretic (Additional file 1: Table S2). The simple regression analysis showed no correlation (beta = − 0.116, 95% CI − 23.7, 12.1, *p* = 0.514), so further adjustment was unnecessary.

## Discussion

The present study assessed the impact of diuretics in managing gout in an inception cohort of patients in specialised care. This issue is a subject of debate in the literature, with conflicting data in the case of allopurinol [10, 14], but we did not observe any significant effect of diuretics on the achievement of SU targets or on the maximum doses prescribed in patients on allopurinol or febuxostat.

Diuretics, especially loop agents and thiazides, are extensively used in clinical practice and are often essential for managing potentially life-threating disorders such as heart or kidney failure. Diuretic-induced hyperuricaemia is a well-established secondary effect and derives from impaired SU excretion due to reducing extracellular volume [15] and interactions with renal urate transport at the proximal convoluted tubule [16]. Diuretics are common in patients with gout, who tend to be older and suffer from a number of comorbidities [17]. In the present study, more than 40% of the study population was on diuretics at baseline. However, whether the development of gout relates to the diuretic-induced hyperuricaemia or is attributable to the underlying disease is still unclear [18].

The dosage of allopurinol needed to achieve SU targets varies widely between patients, depending on variables such as kidney function, body size, or genetic factors influencing tubular reabsorption [19]. Concomitant use of diuretics was shown to increase the doses of allopurinol necessary to reach certain SU levels by 25% to 100% [10]. However, in our adjusted analysis there were no differences in allopurinol dosage based on concomitant diuretic therapy. Wright et al. analysed data from five clinical trials, while here patients were recruited in a clinical practice setting, which is often more complex due to the prevalence of comorbidities. Loop diuretics and thiazides were analysed together in both studies, with no stratification according to dosage in the present study due to the risk of an underpowered analysis. For furosemide, some authors have suggested that allopurinol interferes with down-regulation of xanthine oxidase, explaining higher dosage requirements [20], but this hypothesis has not been fully confirmed [21]. In contrast, no authors have observed significant interactions with thiazides [22, 23]. Further studies should assess the existence of a differential effect in allopurinol dosage between types of diuretics.

Regarding the impact of diuretics on the effect of febuxostat, hydrochlorothiazide showed no significant clinical interaction [24], and we did not identify any studies assessing loop agents from the current literature. The present study showed no differences in dosage or achievement of SU targets based on the use of diuretics. These findings - the first to come from clinical practice - would lend support to the use of febuxostat in this setting. However, they should be interpreted with caution, as our sample included few participants taking this drug, and moreover, febuxostat is only marketed in Europe as 80 mg and 120 mg tablets (thus limiting its dosage, unlike allopurinol). Larger studies are needed to corroborate our results.

Almost a quarter of the patients in the study sample had their diuretics discontinued during follow up. Although this treatment strategy is in line with recommendations from the European League Against Rheumatism (EULAR) [5], these were based on low-quality evidence, and our results do not support this course of action, at least in the case of urate-lowering therapy. Nevertheless, the approach may be of interest when the patient reaches persistent normouricaemia and there is a clinical presumption of complete removal of crystal deposits. ULD dose reduction or discontinuation should aim to maintain uricaemia below its saturation point to prevent new crystal formation [25]. At this point, the absence of factors contributing to hyperuricaemia, such as diuretics, is desirable. However, this research question, while of active interest, was beyond the scope of the present study.

The main strength of this study is its structured assessment of outcomes in a cohort of patients with crystal-proven gout. We analysed a significant sample size of more than 200 patients treated with allopurinol, compared to 133 patients included in the study by Wright et al. [9], allowing a proper evaluation of outcomes in the allopurinol group. Furthermore, we considered several covariates, adjusting the analysis to reinforce the robustness of the results. Regarding the limitations, these mainly relate to the cohort study design and include the absence of random sampling and allocation to ULDs or diuretics, along with the impossibility of modifications to the treatment regimens by researchers. Around 20% of patients included in the inception cohort were excluded from this analysis due to loss of follow up from clinics, a phenomenon that often occurs in clinical practice [26]; however, we analysed complete data in around 250 patients, limiting the impact of these losses. We did not undertake a differential analysis on the type of diuretic, owing to small numbers in the subgroups. Although the results are comparable with current evidence, this should be properly assessed in the future. The effect of diuretics on the duration of achieving the recommended SU targets could provide an additional idea of their impact on management; unfortunately, this issue could not be assessed in the present study as records review focused on the time of maximum dose of ULD prescribed - the primary study outcome. We did not assess safety.

## Conclusion

Both loop agents and thiazides are diuretics that are commonly prescribed in patients with gout and have often been associated with impaired response to allopurinol and refractoriness, prompting recommendations for their discontinuation when possible. However, the results of the present study do not support prior observations related to the achievement of SU targets or the necessary ULD dosage, while they are in keeping with those recently reported by Kannangara et al. [14]. These findings require further confirmation through prospective, intervention studies.

## Additional file

Additional file 1: **Table S1.** Type and dosage of diuretics. **Table S2.** Comparison of maximum dose of urate-lowering drugs regarding the type of diuretics. (DOCX 15 kb)

## Abbreviations

BMI: Body-mass index
CKD: Chronic kidney disease
CKD-EPI: Chronic kidney disease epidemiology collaboration
IQR: Interquartile range
MSU: Monosodium urate
SD: Standard deviation
SU: Serum urate
ULD: Urate-lowering drugs
