# Supplementary material for: Impact of diuretics on the urate lowering therapy in patients with gout: analysis of an inception cohort
Source: Arthritis Res Ther. 2018 Mar 22;20:53. doi: 10.1186/s13075-018-1559-2 (PMC5863897; doi:10.1186/s13075-018-1559-2)
Supplement: Supplementary file 1 — Table S1. Type and dosage of diuretics. Table S2. Comparison of maximum dose of urate-lowering drugs regarding the type of diuretics. (DOCX 15 kb) [file 13075_2018_1559_MOESM1_ESM.docx]

**Impact of diuretics on the urate lowering therapy in patients with gout: analysis of an inception cohort.**

**__ Supplementary material __**

**Table S1. Type and dosage of diuretics.**

| **Baseline** | | | **At maximum dose of ULD** | | |
| --- | --- | --- | --- | --- | --- |
| *Type* | *Dose (mg/day)* | *N (%) [n=106]* | *Type* | *Dose (mg/day)* | *N (%) [n=90]* |
| Hydrochlorothiazide | 12.5  25  50  80  100 | 29 (27.3)  22 (20.8)  6 (5.7)  1 (0.9)  1 (0.9) | Hydrochlorothiazide | 12.5  25  50  100 | 20 (22.2)  14 (15.6)  5 (5.6)  1 (1.1) |
| Chlorthalidone | 100 | 1 (0.9) | Chlorthalidone | 100 | 1 (1.1) |
| Furosemide | 40  60  80  120  160  200 | 13 (12.3)  2 (1.9)  12 (11.3)  2 (1.9)  3 (2.8)  3 (2.8) | Furosemide | 10  40  60  80  120  160  200 | 1 (1.1)  15 (16.7)  3 (3.3)  11 (12.2)  3 (3.3)  4 (4.4)  2 (2.2) |
| Torasemide | 5  10 | 5 (4.7)  5 (4.7) | Torasemide | 5  10 | 8 (8.9)  3 (3.3) |
| Indapamide | 0.625 | 1 (0.9) |  |  |  |

*ULD: urate-lowering drug.*

**Table S2. Comparison of maximum dose of urate-lowering drugs regarding the type of diuretics.**

|  | Thiazide | Loop | Combination | p |
| --- | --- | --- | --- | --- |
| Maximum dose of allopurinol (mg/d) | 300 (200-300) | 300 (113-300) | 150 (100-225) | **0.049** |
| Maximum dose of febuxostat (mg/d) | 80 (80-80) | 80 (40-80) | 100 (80-100) | 0.136 |

Data shown as median (p25-75). Subgroup analyses performed by Kruskal-Wallis’s H test.
